# Understanding the Drying Behavior of Regenerated Cellulose Gel Beads: The Effects of Concentration and Nonsolvents

**DOI:** 10.1021/acsnano.1c09338

**Published:** 2022-02-01

**Authors:** Hailong Li, Margarita Kruteva, Martin Dulle, Zhen Wang, Katarzyna Mystek, Wenhai Ji, Torbjörn Pettersson, Lars Wågberg

**Affiliations:** † Department of Fibre and Polymer Technology, KTH Royal Institute of Technology, Teknikringen 58, SE-100 44 Stockholm, Sweden; ‡ Department of Physics, AlbaNova University Center, Stockholm University, 10691 Stockholm, Sweden; § Jülich Centre for Neutron Scattering and Biological Matter (JCNS-1/IBI-8), Forschungszentrum Jülich GmbH, Wilhelm-Johnen-Straße, D-52425 Jülich, Germany; ⊥ Deutsches Elektronen-Synchrotron (DESY), Notkestr. 85, 22607 Hamburg, Germany; ¶ Wallenberg Wood Science Centre, Department of Fibre and Polymer Technology, KTH Royal Institute of Technology, Teknikringen 56, 10044 Stockholm, Sweden

**Keywords:** regenerated cellulose, gel bead, drying kinetics, nonsolvent, cellulose concentration

## Abstract

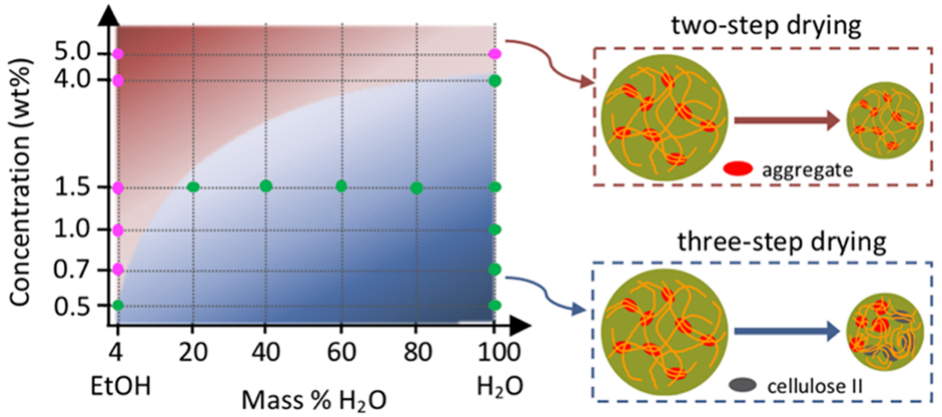

The drying behavior of regenerated cellulose gel beads swollen with different nonsolvents (*e.g*., water, ethanol, water/ethanol mixtures) is studied *in situ* on the macroscopic scale with an optical microscope as well as on nanoscale using small-angle/wide-angle X-ray scattering (SAXS/WAXS) techniques. Depending on the cellulose concentration, the structural evolution of beads during drying follows one of three distinct regimes. First, when the cellulose concentration is lower than 0.5 wt %, the drying process comprises three steps and, regardless of the water/ethanol mixture composition, a sharp structural transition corresponding to the formation of a cellulose II crystalline structure is observed. Second, when the cellulose concentration is higher than 5.0 wt %, a two-step drying process is observed and no structural transition occurs for any of the beads studied. Third, when the cellulose concentration is between 0.5 and 5.0 wt %, the drying process is dependent on the nonsolvent composition. A three-step drying process takes place for beads swollen with water/ethanol mixtures with a water content higher than 20%, while a two-step drying process is observed when the water content is lower than 20%. To describe the drying behavior governed by the cellulose concentration and nonsolvent composition, a simplified phase diagram is proposed.

Cellulose is one of the most abundant natural polymers on earth and has been used in a variety of applications due to its excellent physical, mechanical, and biocompatible properties.^1−3^ High-performance products such as high tenacity rayon,^4,5^ transparent films,^6−8^ hydrogels and aerogels,^9−11^ as well as spheres and beads^12^ can be fabricated by the regeneration of cellulose solutions into materials that can be used in dry state where they exhibit good strength and toughness. The mechanism of cellulose dissolution has been intensively studied using several different solvent systems, notably mixtures of lithium chloride and *N*,*N*-dimethylacetamide (LiCl/DMAc),^13−18^ which is used in this work. However, a limited number of studies have investigated the kinetics of the microstructural evolution during the drying of regenerated cellulose.^19^ This is a crucial step in shaping cellulose materials from the wet state; it is essential to determine how the micro- or nanoscale structures change during the removal of water or other regenerating solvents. To simplify these studies, it is important to prepare well-defined model systems for the cellulose-based materials. It has already been shown that cellulose gel beads, which are smooth on the nanoscale and can be prepared by precipitating the cellulose solution into a nonsolvent (ethanol or water), can be used as a suitable model system.^20−25^ This is mostly due to the fact that it has been possible to accurately characterize the highly homogeneous structure of cellulose beads and show that they are composed of a noncrystalline, molecularly dispersed cellulose network.^21,22^ Such systems have already been used to investigate the swelling behavior of wet, delignified cellulosic wood fibers;^21,22^ the adhesion of two cellulose surfaces;^20,25^ and the influence of polymer grafting on the adhesion of modified beads on a molecular scale.^23^ However, the structural developments during drying from different solvents and for different starting concentrations of the cellulose solution are still unexplored research areas.

X-ray scattering is a powerful technique used to investigate the micro structure of almost every kind of material including cellulose.^26^ For example, grazing incidence small-angle X-ray scattering (GISAXS) has been used to investigate supramolecular rearrangements in cellulose thin films during the conversion of trimethylsilyl cellulose to cellulose via HCl vapor hydrolysis.^27,28^ GISAXS was also employed by Roth et al. to characterize the structure of spray-deposited nanocellulose thin films and water-induced structural rearrangements during drying.^29,30^ In our previous work, small-angle and wide-angle X-ray scattering (SAXS and WAXS) methods were utilized to trace the structural evolution of 1.5 wt % regenerated cellulose gel beads (swollen in water or ethanol) during drying.^24^ Likewise, GISAXS in combination with AFM was used to trace the structural evolution of cellulose-cellulose interfaces joining together during drying.^25^ All these efforts, performed to clarify the molecular interactions at cellulose interfaces during drying, have had the common objective to quantify the relative importance of the different molecular interactions that are active between cellulose surfaces in the wet and dry state. Despite the common use of cellulose-based materials, such as paper, packaging products, hygiene materials, and high value-added nonwoven materials, it is surprisingly still not known which forces are holding cellulose materials together. Vague arguments about the dominating influence of hydrogen bonding have been filling the literature, and a recent review^31^ has summarized the current knowledge about the importance of different types of molecular interactions. The lack of well-defined cellulose model materials has been, and still is, an important factor behind the lack of clear model experiments where the influence of different interactions can be clarified. The development of nanometer, smooth, and well-defined cellulose beads is essential in this work since they are suitable for new, high-resolution measuring techniques. The understanding of these interactions will also be even more important in a future biobased society where water-based processes and renewable materials will be necessary. Furthermore, by combining different types of forms of cellulose and also cellulose and other materials in these beads, they might also form the base for new types of biocomposite materials.^32^ In the present work, the objective was to explore how the cellulose gel beads dry when different cellulose concentrations and nonsolvent compositions are used. Consequently, a comprehensive evaluation of the microstructural changes of cellulose gel beads clarifies how cellulose surfaces consolidate during the drying of cellulose-rich materials.

In this study, cellulose gel beads were prepared using different cellulose concentrations and swollen by different nonsolvents: water, ethanol, and water/ethanol mixtures, as depicted in Figure 1a. The change in diameter of the beads was recorded with an optical microscope during drying (Figure 1b). The micro- or nanoscale structural evolution during drying was investigated using *in situ* SAXS and WAXS techniques. Additionally, the drying behavior of cellulose gel beads is discussed and illustrated in a simplified phase diagram.

**Figure 1:**
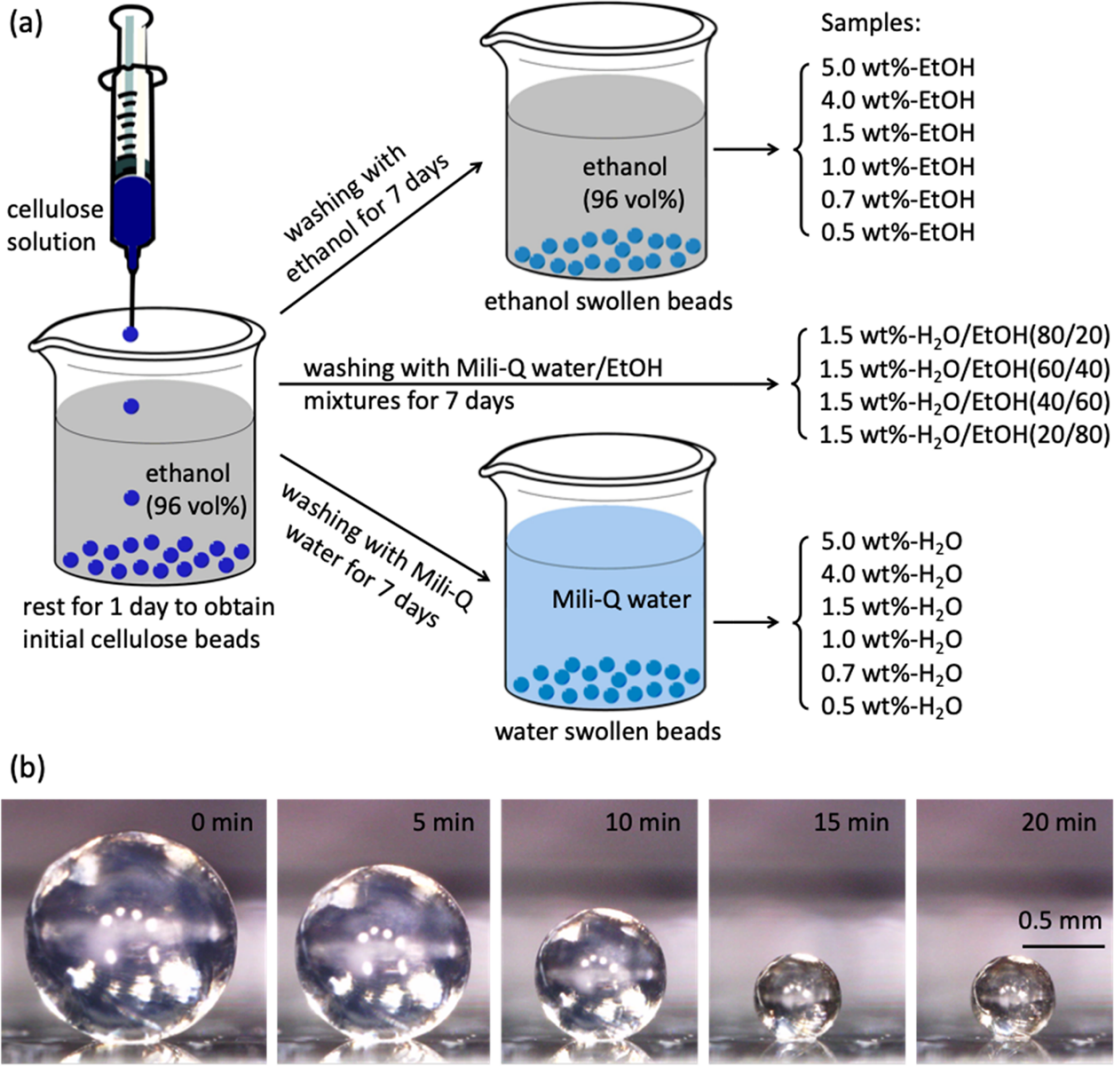
(a) Schematic illustration of the preparation of the cellulose gel beads swollen in water, ethanol, and water/ethanol mixtures. All the samples are listed in the panel on the right-hand side. (b) Side-view optical microscope images of cellulose gel bead (1.5 wt %-H_2_O) during drying on a glass slide at 22 °C and 28% RH. The scale bar corresponds to a length of 0.5 mm for all the images.

## Results and Discussion

### Interior Morphologies of the Initial Cellulose Gel Beads

**Figure 2:**
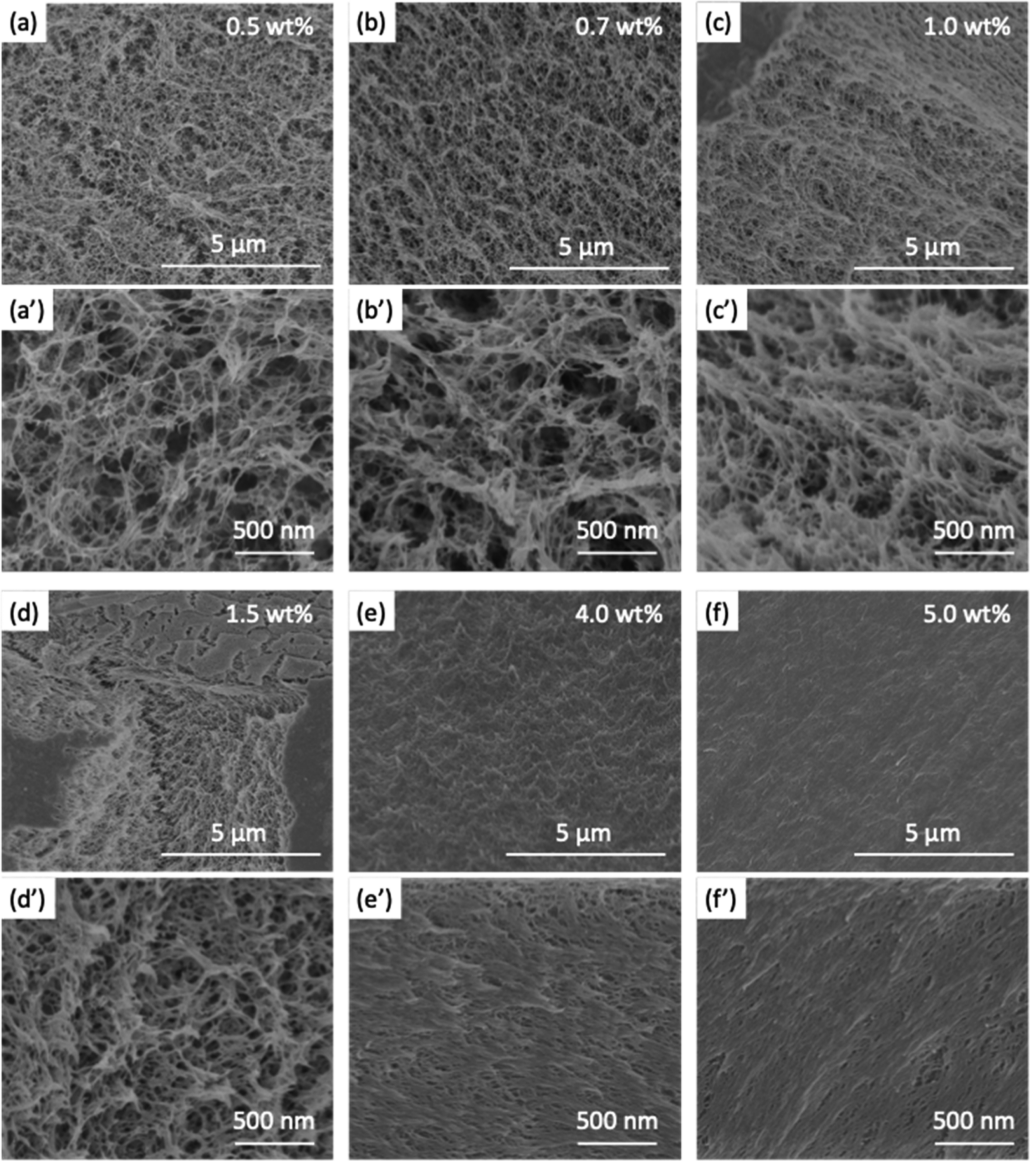
SEM images of the interior of CPD dried ethanol swollen cellulose beads prepared using different cellulose concentrations: (a, a′) 0.5 wt %-EtOH, (b, b′) 0.7 wt %-EtOH, (c, c′) 1.0 wt %-EtOH, (d, d′) 1.5 wt %-EtOH, (e, e′) 4.0 wt %-EtOH, and (f, f′) 5.0 wt %-EtOH. (a–g) are lower magnification SEM images and (a′-g′) are higher magnification SEM images.

The scanning electron microscopic (SEM) images of the internal structure of critical point dried (CPD) ethanol swollen beads are shown in Figure 2. A porous 3D network composed of fibrillar cellulose is observed for all types of CPD beads. This porous structure is more obvious for the low cellulose concentration beads such as the 0.5 and 0.7 wt % beads (Figure 2a′,b′). As the cellulose concentration increases, the porous structure becomes denser since the cellulose fibrils are thicker, which causes a decrease in pore size (Figure 2a–d, 2a′–d′). When the cellulose concentration is 4 wt % or higher, the fibrillar networks are more compact, resulting in the pores becoming smaller and harder to observe (Figure 2e,f and 2e′,f′). Similar porous structure is observed from the SEM images of the interior of CPD dried water swollen beads (Figure S1), and the trend is consistent with ethanol swollen beads. For 1.5 wt % cellulose gel beads swollen by different water/ethanol mixtures, the porous structure becomes slightly denser when the water content in the water/ethanol mixture increases (Figure S2). It is expected to be caused by the large interaction between water and cellulose.

### Drying Behavior of Cellulose Gel Beads: Microscopy

**Figure 3:**
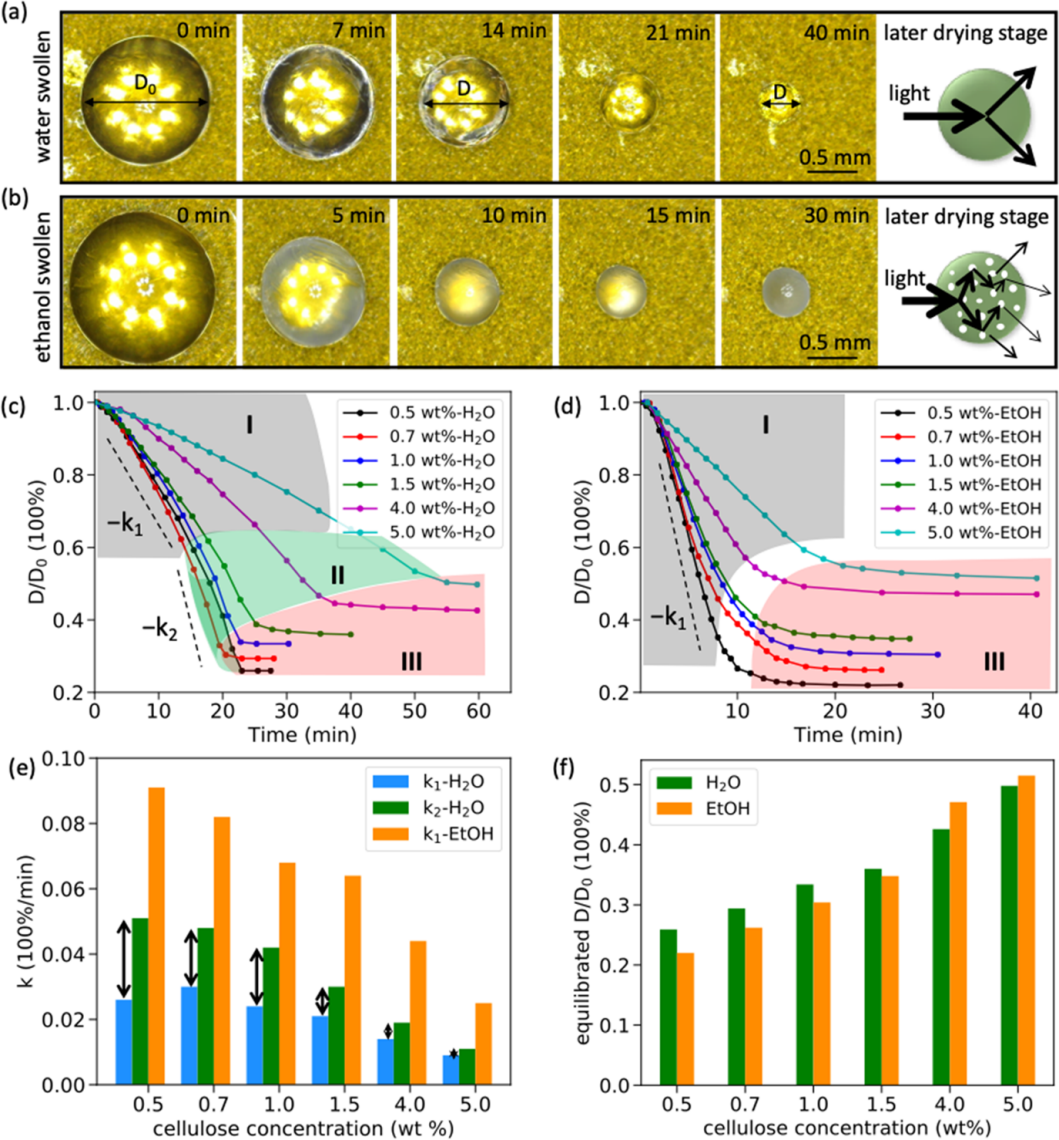
Representative top-view optical microscope images of (a) 1.0 wt %-H_2_O and (b) 1.0 wt %-EtOH beads during drying on Kapton tape at 26 °C and 33% RH (scale bars are 0.5 mm for all images). The normalized diameter *D*/*D*_0_ for (c) water swollen and (d) ethanol swollen beads during drying. (e) Linear shrinking rate (*k*_1_ and *k*_2_) in drying phases I and II. (f) Equilibrated *D*/*D*_0_*vs* cellulose concentration for water swollen and ethanol swollen beads.

From the side-view optical microscope images captured during drying of a cellulose gel bead (Figure 1b), it can be seen that the swollen bead shrinks continuously and uniformly, retaining its spherical shape during the drying process. This is consistent with our previous work.^24,33^ Moreover, apart from the size decrease, the transparency of the gel beads changes with evaporation, as shown in Figure 3a and b. For the water swollen bead (1.0 wt %-H_2_O, in Figure 3a), the outer part becomes translucent during the first 7 min and then becomes more transparent for the following 14 min. After that, the entire bead remains transparent, up to and including when it is completely dry. The same phenomenon is observed for water swollen beads formed using other cellulose concentrations (Figure S3). In contrast, in the case of the ethanol swollen beads (1.0 wt %-EtOH, in Figure 3b), the outer part becomes opaque in the first 5 min and the bead never recovers its transparency, remaining opaque and gray when dried for 30 min. This phenomenon is also observed in other studies of ethanol swollen beads formed with different cellulose concentrations (Figure S4). This is most likely due to the multiscattering and reflection of light inside the ethanol swollen beads, which have a tendency to keep their porous internal structure during drying, as established in our previous work.^24^ For the water swollen beads, the fibrillar-like structure in the drying bead is compacted and the light scattering pores, initially present during drying, are removed to such an extent that a transparent dry bead is formed. Schematic figures are provided on the right of Figure 3a and b to illustrate this.

Figure 3c and d show the diameter changes of the water swollen and ethanol swollen beads during the drying process, respectively, where the *D*/*D*_0_ values are the diameter of the bead (*D*) normalized to the initial diameter (*D*_0_). For the water swollen beads, *D*/*D*_0_ decreases linearly at short evaporation times (stage I, gray regime in Figure 3c), and the linear shrinking rate (*k*_1_) decreases with the cellulose concentration after being relatively constant for the two lowest concentrations. After the first stage of drying, a fast linear shrinking with a higher rate, *k*_2_, occurs, indicating that the drying stage II has begun (green regime in Figure 3c). This faster shrinking is more pronounced for low cellulose concentration gel beads, which have a shorter residence time in stage I drying. After drying for a certain time, *D*/*D*_0_ starts to become constant (red regime in Figure 3c), which is defined as the “equilibrated *D*/*D*_0_”. The ethanol swollen beads exhibit a different drying behavior, where only one linear shrinking phase is observed (Figure 3d) before reaching the equilibrated *D*/*D*_0_. These results support the proposed hypothesis that the light scattering pores, which are initially present in the internal structure of gel beads, are shrinking for water swollen beads but are retained throughout the drying process of ethanol swollen beads. The reasons for this are discussed in more detail below with the results of the SAXS experiments.

Figure 3e summarizes the linear shrinking rate (*k*_1_ and *k*_2_) in the drying stages I and II *vs* cellulose concentration for water swollen and ethanol swollen beads. For water swollen beads, with the exception of the 0.5 wt %-H_2_O bead, both *k*_1_ and *k*_2_ decrease as cellulose concentration increases. The rate difference between *k*_2_ and *k*_1_ is reduced for samples with higher cellulose concentrations. For the 5 wt %-H_2_O beads, *k*_2_ is almost the same as *k*_1_, which means that the faster shrinking during drying stage II is not as pronounced as with the lower concentration cellulose beads. In the case of ethanol swollen beads, it is observed that *k*_1_ decreases as cellulose concentration increases. Furthermore, the values of *k*_1_ are significantly larger for the ethanol swollen beads than the *k*_1_ values of water swollen beads with the same cellulose concentration, which is most likely due to the faster evaporation rate of ethanol (due to its lower vapor pressure) at ambient conditions.

Figure 3f shows the change in equilibrated *D*/*D*_0_*vs* cellulose concentration for water swollen and ethanol swollen beads. It can be seen that the *D*/*D*_0_ value increases with higher cellulose concentrations for both water swollen and ethanol swollen beads, indicating that higher cellulose concentrations better preserve the initial bead size. Interestingly, for cellulose concentrations lower than or equal to 1.5 wt %, the equilibrated *D*/*D*_0_ values of ethanol swollen beads are smaller than those for the corresponding water swollen beads. This would suggest that these ethanol swollen beads shrink more than their water swollen bead counterparts. However, when higher cellulose concentrations are used (4.0 and 5.0 wt %), the equilibrated *D*/*D*_0_ values determined for ethanol swollen beads are higher than those of the corresponding water swollen beads, meaning that the ethanol swollen beads shrink less. This phenomenon, together with the aforementioned slower shrinking rate (*k*_1_) for the 0.5 wt %-H_2_O bead, are linked to the microscale structural evolution of beads during drying from different solvents (discussed further in the Wide-Angle X-ray Scattering (WAXS) section below).

### Structural Evolution of Gel Beads during Drying: Small-Angle X-ray Scattering (SAXS)

To complement the studies of the bead drying on the macroscopic scale, SAXS was used, as depicted in Figure S5a, to study structural changes on the microscale of water swollen and ethanol swollen beads during drying. Representative 2D SAXS patterns for 1.5 wt %-H_2_O beads are presented in Figure S5b. The homogeneous scattering patterns indicate an isotropic structure of the cellulose gel bead network throughout the drying process, which is consistent with the aforementioned results that showed the gel bead retaining its spherical shape during drying (Figure 3a,c and Figure 1b). The scattering intensity increases in the early drying stage (<20.5 min) and then drops to a significantly lower value over the following 2 min before staying constant.

**Figure 4:**
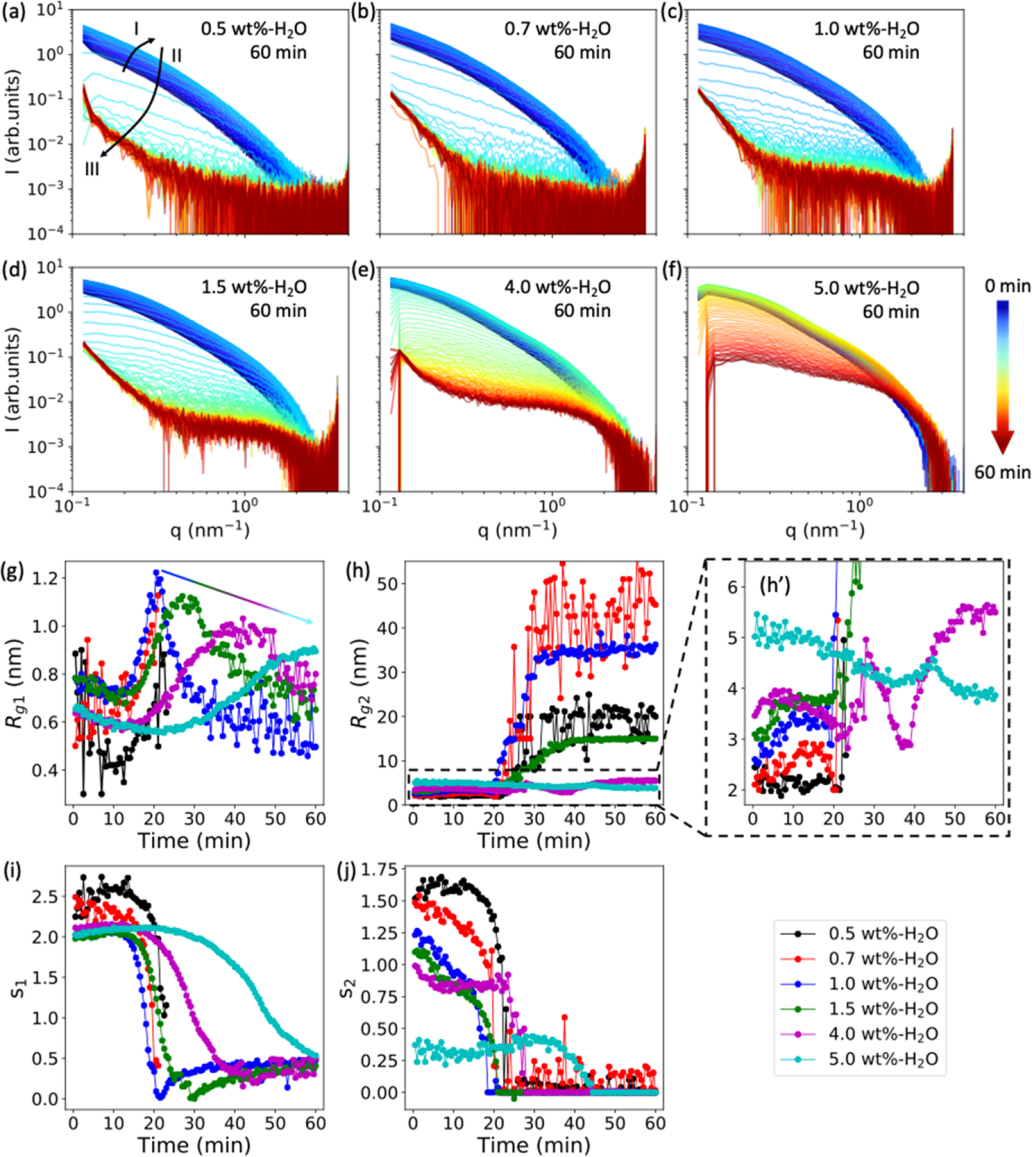
(a–f) SAXS curves for 0.5, 0.7, 1.0, 1.5, 4.0, and 5.0 wt %-H_2_O beads measured during drying over 60 min, with a time step of 30 s between each curve. The color arrow bar indicates the drying time from 0 min (blue) to 60 min (red). (g–j) Change in the fitted length scale (*R*_*g*1_ and *R*_*g*2_) and “dimensionality” parameters (*s*_1_ and *s*_2_) as a function of the drying time.

Figure 4a–f show 1D SAXS curves for all water swollen beads throughout the drying process, achieved through an integration over the full azimuthal angle range of the 2D SAXS patterns. Similar to the *D*/*D*_0_*vs* time curves shown in Figure 3c, three regimes can be distinguished in the SAXS curves for water swollen beads (as indicated by arrows and roman numerals for the different phases of the 0.5 wt %-H_2_O bead shown in Figure 4a). In regime I, the shape of SAXS curves does not change significantly, although the intensity increases with evaporation time due to more X-rays being scattered by objects in the beads during shrinking. After drying for around 20 min (regime II), the intensity of the SAXS curves decreases rapidly as the evaporation time increases, and the curves start to bend up in the low *q* region, which indicates that a potential structural change is occurring during this time period. In the later drying phase (regime III), the SAXS curves remain relatively unchanged, and consequently, it is assumed that there is no structural change in the beads in this regime. These three drying regimes were observed in all water swollen beads with a cellulose concentration of 4.0 wt % or less (Figure 4a–e). However, for the 5.0 wt %-H_2_O bead (Figure 4f), the SAXS curve does not bend upward in the low *q* region in the final drying phase, unlike the lower cellulose concentration beads.

To quantitatively evaluate the microscale structural changes of the cellulose beads, a Guinier-Porod model^34^ was used to consistently fit the SAXS data collected during the drying process. In this model, two characteristic length scales (*R*_*g*1_ and *R*_*g*2_) and corresponding “dimensionality” parameters (*s*_1_ and *s*_2_) are defined to characterize the shape of the objects present within the system. The fit functions and analysis procedure are summarized in detail in the literature^35,36^ as well as in our previous work.^24^ Briefly, *R*_*g*1_, in the size range of the anhydroglucose unit, is related to the local cellulose monomer, and *R*_*g*2_ corresponds to the size of the elongated aggregate structures of the anhydroglucose units inside the gel beads. The shape of the structures can be determined by *s*_1_ and *s*_2_: when *s*_1_ = *s*_2_ = 0, the scattering object has a spherical symmetry; if *s*_1_ = 1 and *s*_2_ = 0, it has a cylindrical shape; and when *s*_1_ = 2 and *s*_2_ = 0, it corresponds to a lamellae structure with equal width and length. However, the real-world microscopic structure of the cellulose beads is nonideal and complex, especially during drying, we suggest that it is best represented by a combination of the aforementioned structures. Figure S6 shows raw data measured during the drying of a 1.0 wt %-H_2_O bead and the corresponding fitting curves.

Figure 4g–j summarize the *R*_*g*1_, *R*_*g*2_, *s*_1_, and *s*_2_ values of all the water swollen beads throughout the drying process. As with the macroscopic drying behavior observed in Figure 3c, the evolution of *R*_*g*1_ values can be divided into three regimes (Figure 4g). For 1.0 wt %-H_2_O swollen beads (blue curve in Figure 4g), the value of *R*_*g*1_ decreases from 0.78 to 0.71 nm during the first 12 min (regime I) and then quickly increases to 1.2 nm during the next 8 min (regime II), after which it decreases to 0.6 nm in 15 min before steadily decreasing to 0.5 nm (regime III). For beads with cellulose concentrations of 1.0 wt % and higher, all the water swollen beads have similar *R*_*g*1_*vs* time curves with three regimes. The exception to this is the 5.0 wt %-H_2_O beads on which no regime III was observed due to not practically taking the measurements over a sufficiently long period of time for these samples to dry to this stage. At higher cellulose concentrations, the *R*_*g*1_*vs* time curves are stretched for each regime due to the slower drying rate of these beads. The maximum value of *R*_*g*1_ decreases from 1.2 to 0.9 nm when the cellulose concentration is increased from 1.0 wt % to 5.0 wt %. In regime III, the *R*_*g*1_ decrease is slower, with a higher value at 60 min for higher concentration cellulose beads. For the 0.5 wt %-H_2_O and 0.7 wt %-H_2_O beads, similar trends in the curves are observed. However, the SAXS curves are quite noisy in the high *q* region in the later drying phase (regime III in Figure 4a,b) and the extracted values for *R*_*g*1_ and *s*_1_ after a drying time of 22 min are therefore less reliable and are not plotted in Figure 4g and i but have been included for reference in Figure S7. As predicted from the aforementioned *R*_*g*1_ evolution of gel beads with high cellulose concentrations, a much faster decrease and lower values of *R*_*g*1_ in regime III are observed when beads are made with lower cellulose concentrations, although the extracted values have slightly larger errors.

The aggregate structure (*R*_*g*2_) size in 1.0 wt %-H_2_O beads (blue curve in Figure 4h,h′) increases from 2.50 to 3.25 nm during the first 12 min of drying (regime I), after which it remains constant during the next 8 min of drying (regime II), before increasing to 32.00 nm over the next 15 min, and reaching 36.0 nm after a final 25 min of drying (regime III). Although the diameter of the gel bead decreases minimally in regime III (see Figure 3c), a sharp increase of *R*_*g*2_ is observed here, indicating that the nanoscale structures change significantly for 1.0 wt %-H_2_O beads during the later drying phase. As proposed in our previous work,^24^ the rapid increase of *R*_*g*2_ is related to a sharp structural transition caused by the collapse of the nanoporous structure.

A similar trend and sharp increase of *R*_*g*2_*vs* time are observed for 0.5, 0.7, 1.5, and 4.0 wt %-H_2_O beads, while for 5.0 wt %-H_2_O beads, there is no sharp increase in *R*_*g*2_. Figure 4h′ shows that *R*_*g*2_ for the 5.0 wt %-H_2_O bead decreases from 5.2 to 4.1 nm in the first 35 min before slightly increasing to 4.6 nm over the next 10 min, and finally decreasing to 3.9 nm in the later drying phase. Interestingly, *R*_*g*2_ shows higher values for increasing cellulose concentrations before drying (Figure 4h′) but shows lower values for increasing cellulose concentrations after 60 min of drying, with the exception of the 0.5 wt %-H_2_O bead (Figure 4h).

**Figure 5:**
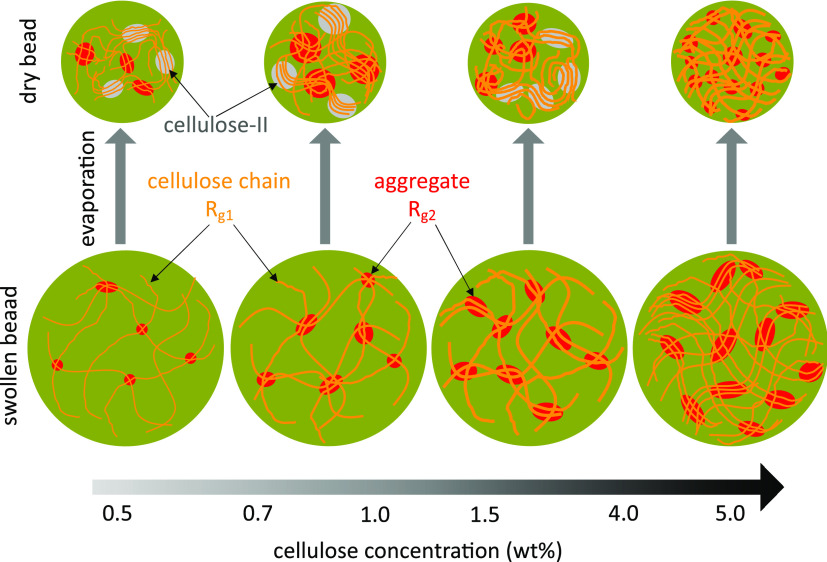
Illustration of the structural evolution of water swollen beads during water evaporation from cellulose gel beads with different cellulose concentrations, based on SAXS/WAXS measurements.

The *R*_*g*1_, *R*_*g*2_, and shape factors indicate that aggregates are formed by entanglements of the cellulose chains in the presence of nonsolvents as they evaporate. The size changes of these aggregates are illustrated in Figure 5. In regime I, the number of entanglements increases as cellulose concentrations increase, which leads to the formation of many large aggregates. As a consequence, the gel beads become much stronger and more resilient to the collapse of the porous structure. This is observed on the 5.0 wt %-H_2_O bead as an absence of the sharp increase in *R*_*g*2_. For the 0.5 wt %-H_2_O bead, the final value of *R*_*g*2_ is about 20 nm, which is smaller than the values observed for the 0.7 and 1.0 wt %-H_2_O beads. We suggest that the probable reason for this is that the very low cellulose concentration prevents the formation of aggregates inside the 0.5 wt %-H_2_O bead. This explanation is consistent with the lower shrinking rate (*k*_1_) measured for the 0.5 wt %-H_2_O bead compared with the 0.7 and 1.0 wt %-H_2_O beads (Figure 3e). Figure 4i shows that the “dimensionality” parameter *s*_1_ stays in the range of 2.5–2.0 in regime I, quickly decreasing to 0.5 in regime II, and then staying constant at this value in regime III. However, as observed in Figure 4j, *s*_2_ changes at the beginning of the bead drying, from 1.6 for 0.5 wt %-H_2_O beads to 0.3 for 5.0 wt %-H_2_O beads, before quickly decreasing to 0 in regime II and staying at 0 in regime III. The evolution of *s*_1_ and *s*_2_ suggests that the shape of the aggregates changes from elongated to spherical during the drying process, and the cellulose chains rearrange within low concentration beads into spherical aggregates with cellulose-II crystalline structures (discussed further in the Wide-Angle X-ray Scattering (WAXS) section). Moreover, the initial shapes of the aggregates in regime I are more elongated in beads with higher cellulose concentrations (Figure 5).

**Figure 6:**
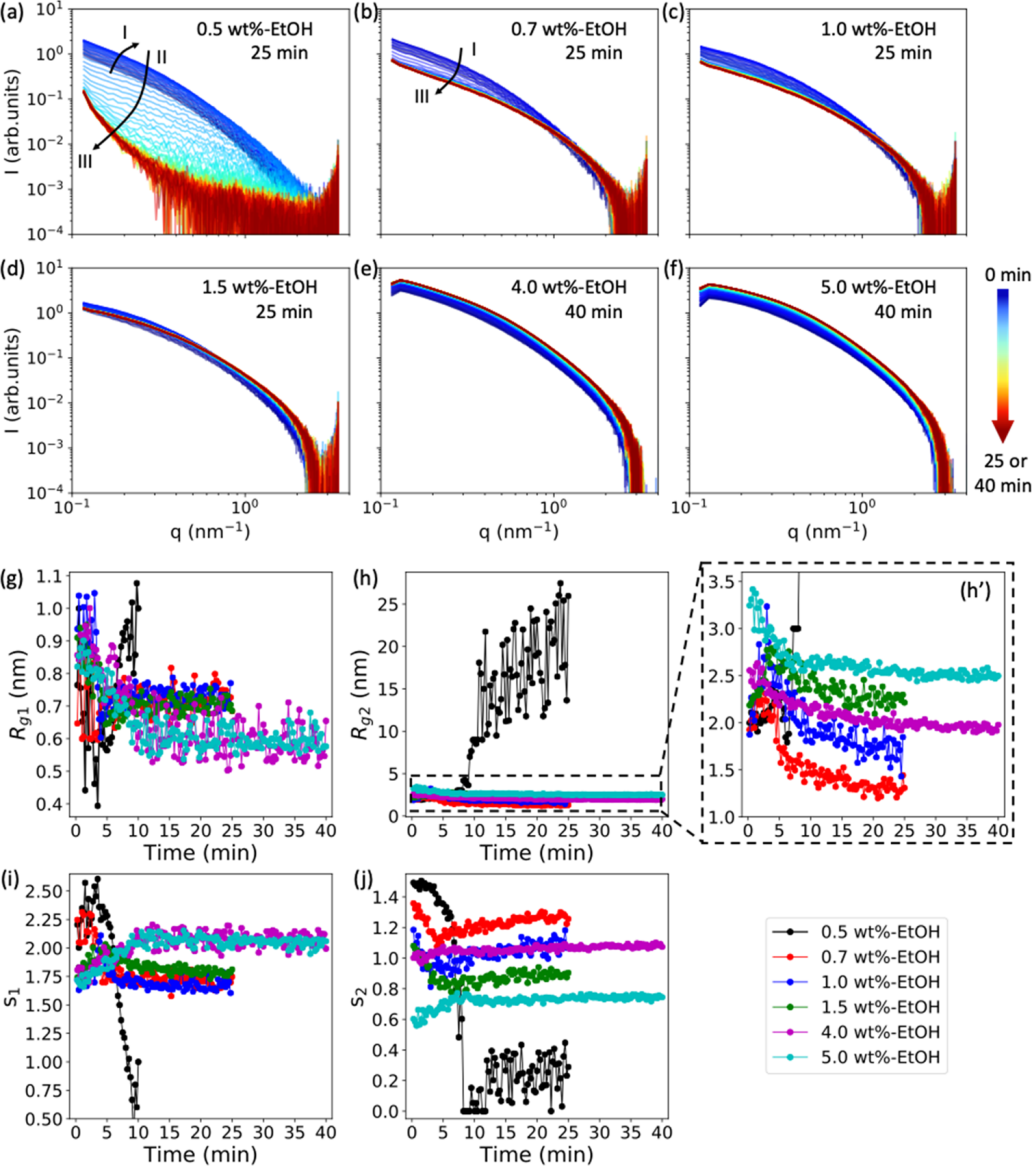
(a–f) SAXS curves for 0.5, 0.7, 1.0, 1.5, 4.0, and 5.0 wt %-EtOH beads measured during drying over a time of 25 or 40 min, with a time step of 15 s between each curve. The colored arrow bar indicates the drying time from 0 min (blue) to 25 or 40 min (red). (g–j) Change in the fitted length scale (*R*_*g*1_ and *R*_*g*2_) and “dimensionality” parameters (*s*_1_ and *s*_2_) as a function of the drying time.

The SAXS measurements were also performed on ethanol swollen beads as they dried, and the extracted 1D SAXS curves are shown in Figure 6a–f. The evolution of the SAXS curves for 0.5 wt %-EtOH beads shown in Figure 6a are similar to the ones for the 0.5 wt %-H_2_O beads shown in Figure 4a. However, when the cellulose concentration is 0.7 wt % or higher, the three drying regimes observed for water swollen beads are not observed in ethanol swollen beads (Figure 6b–f). The shape of the SAXS curves does not vary significantly during the drying process, except for in the early drying phase of the 0.7 wt %-EtOH and 1.0 wt %-EtOH beads. The fitted values of *R*_*g*1_, *R*_*g*2_, *s*_1_, and *s*_2_ are summarized in Figure 6g–j. For cellulose concentrations greater than 0.7 wt %, *R*_*g*1_ rapidly decreases from roughly 0.9 nm to approximately 0.6 nm in less than 10 min, after which it remains relatively constant throughout the later drying phase (Figure 6g). A similar trend in *R*_*g*2_ is observed in Figure 6h′. Interestingly, the sharp increase of *R*_*g*2_ observed for water swollen beads is not seen here (Figure 6h′). Thus, the structure of ethanol swollen beads does not change significantly, and the porous structure does not collapse during the evaporation of ethanol for beads with cellulose concentrations of 0.7 wt % or greater, which is consistent with our previous work.^24^ From the change of the “dimensionality” parameters (*s*_1_ and *s*_2_) with drying time, illustrated in Figure 6i and j, the shapes of these structures in ethanol swollen beads with cellulose concentrations of 0.7 wt % and higher are relatively constant when compared to the corresponding water swollen beads. However, for 0.5 wt %-EtOH beads, the trends in *R*_*g*1_, *R*_*g*2_, *s*_1_, and *s*_2_*vs* time (Figure 6h,j and Figure S8) are similar to those of the 0.5 wt %-H_2_O bead, where a sharp increase in *R*_*g*2_ is observed. This indicates that the sharp structural change can also occur in the ethanol swollen bead as long as the cellulose concentration is sufficiently low, for example, 0.5 wt %.

On the basis of the SAXS results, we propose an interpretation of the phenomenon observed in Figure 3f, where equilibrated *D*/*D*_0_ values of 0.5, 0.7, 1.0, and 1.5 wt %-H_2_O beads are larger than the values of the corresponding ethanol swollen beads. Notably, the equilibrated *D*/*D*_0_ values of 4.0 and 5.0 wt %-H_2_O beads are lower than those of 4.0 and 5.0 wt %-EtOH beads. First, there is no structural transition in the 5.0 wt %-H_2_O bead (Figure 4h) or in the 5.0 wt %-EtOH bead. Therefore, when comparing the drying of the 5.0 wt %-EtOH and 5 wt %-H_2_O beads, structural changes are ignored. Second, from our previous atomic force microscopy (AFM) indentation experiments,^33^ we know that the Young’s modulus determined in the early drying phase for the water swollen bead is lower than for an ethanol swollen bead with the same cellulose concentration. This means that the water swollen bead has a higher tendency to deform in the early drying phase. Third, the surface tension of water is 72 mN/m, which is three-times higher than that of ethanol, 22 mN/m.^37^ Given these three facts, it follows logical intuition that 5.0 wt %-H_2_O beads are more easily deformed than 5.0 wt %-EtOH beads. The same is valid for the 4.0 wt % beads, although the size of *R*_*g*2_ for 4.0 wt %-H_2_O bead does increase very slightly from 4 to 5.5 nm in the later drying phase (Figure 4h′). Therefore, the equilibrated *D*/*D*_0_ values of 4.0 and 5.0 wt %-H_2_O beads are smaller than those of 4.0 and 5.0 wt %-EtOH beads. This situation is reversed for the 0.5, 0.7, 1.0, and 1.5 wt %-H_2_O beads for which there are rapid structural changes during drying (Figure 4h). The most probable reason is that the cellulose network structures formed during drying (illustrated in Figure 5) prevent bead deformation and, as a result, the equilibrated *D*/*D*_0_ values of 0.5, 0.7, 1.0, and 1.5 wt %-H_2_O beads are larger than those of the 0.5, 0.7, 1.0, and 1.5 wt %-EtOH beads.

### Wide-Angle X-ray Scattering (WAXS)

**Figure 7:**
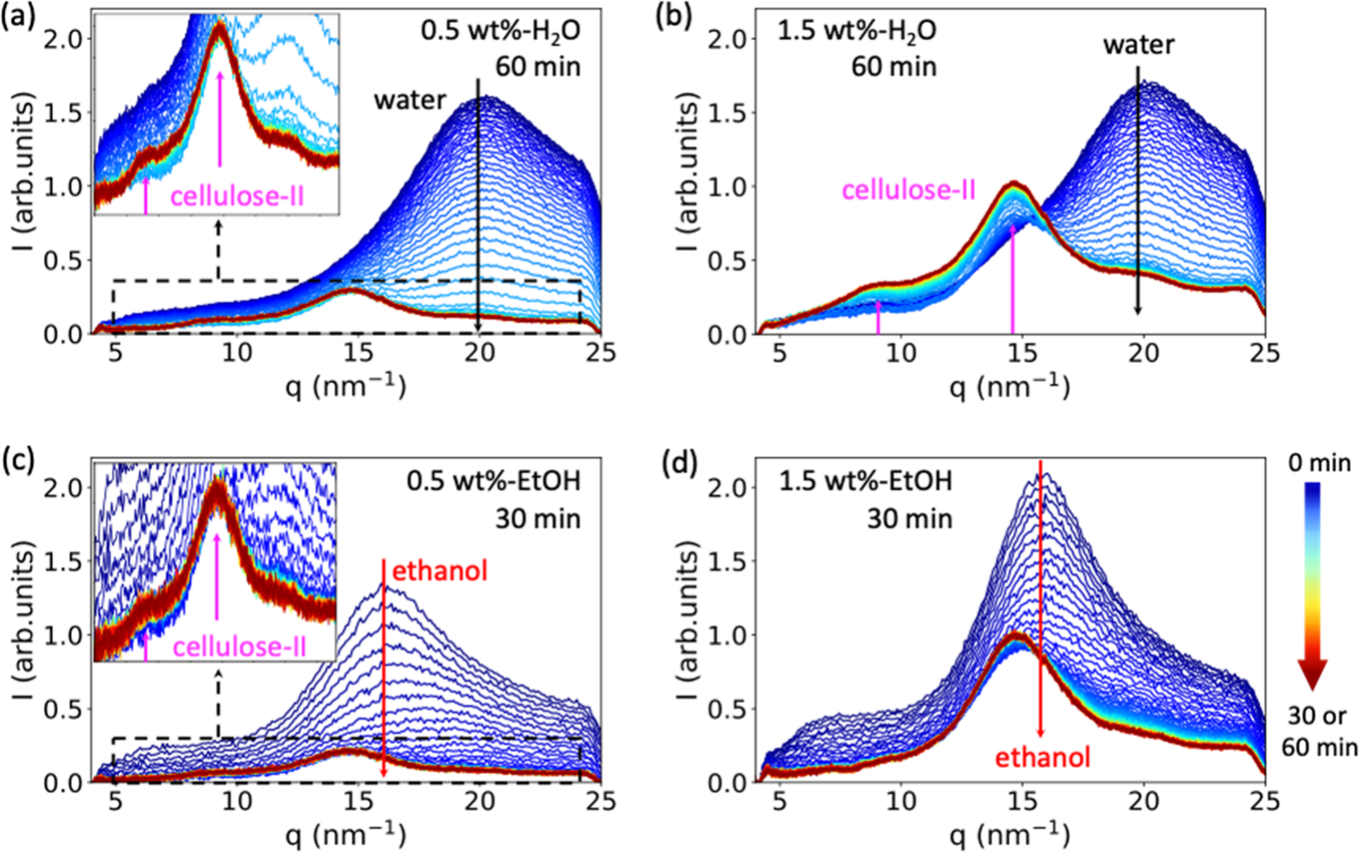
WAXS curves for (a) 0.5 wt %-H_2_O, (b) 1.5 wt %-H_2_O, (c) 0.5 wt %-EtOH, and (d) 1.5 wt %-EtOH beads during the drying process. Water swollen and ethanol swollen beads were dried for 60 and 30 min, respectively. Each curve was measured at 30 and 15 s intervals for water swollen and ethanol swollen beads, respectively. The color arrow bar indicates the drying time from 0 min (blue) to 30 or 60 min (red).

To investigate whether any crystalline order is developed during the drying process, WAXS measurements were conducted for 0.5 and 1.5 wt % beads swollen in water and ethanol; the corresponding WAXS curves measured during the drying process are summarized in Figure 7a–d. According to our previous work^24^ and the literature,^38−41^*q* = 8.8 and 14.6 nm^–1^ (indicated by magenta arrows in Figure 7a–c) are assigned to (11̅0) and (110) crystallographic planes of the cellulose II structure. Then *q* = 20.0 and 15.5 nm^–1^ (indicated by black and red arrows) are assigned to scattering peaks from water and ethanol, respectively.^42,43^ For all the beads, no scattering peaks from the cellulose crystalline structure were observed before drying, which indicated that the swollen beads are amorphous. In the early drying phase, the intensity of the scattering peaks from the nonsolvents (water or ethanol) decreases quickly due to evaporation. After this, the scattering peaks from the cellulose II structure appear and their intensity increases with evaporation time for the 0.5 wt %-H_2_O, 1.5 wt %-H_2_O, and 0.5 wt %-EtOH beads. This means that the crystalline structures (cellulose II) are formed in drying phase II and grow as the drying time increases. In the SAXS results, a sharp increase of *R*_*g*2_, which measures the size of the aggregate structures, was also observed for 0.5 wt %-H_2_O, 1.5 wt %-H_2_O, and 0.5 wt %-EtOH beads in drying phase II. Thus, we can correlate these two phenomena and propose that cellulose chains reorganize into larger crystalline aggregates, as illustrated by the gray domains in Figure 5, during the second drying phase. This hypothesis is further evidenced by the fact that there were no cellulose II scattering peaks for the 1.5 wt %-EtOH bead after drying, as would be expected since there was no sharp increase of *R*_*g*2_ observed in the SAXS measurements of the same bead (Figure 6h). This prediction is supported by the result in Figure 7d, where no scattering peaks attributable to cellulose II structure are observed. According to the proposed hypothesis, the formation time and the size of crystalline domains for different gel beads can be obtained from Figure 4h and Figure 6h. For example, for 0.7 wt %-H_2_O beads, the crystalline domain starts to form after the first 20 min’s drying and its size increases to ∼40 nm in 10 min. While, for 1.5 wt %-H_2_O beads, after the first 20 min’s drying the crystalline domain starts to form and its size increases to 20 nm in 20 min. Therefore, the formation time and size of the crystalline domains for different cellulose gel beads are determined by both cellulose concentration and nonsolvents. Note, due to the strong scattering of solvents, the change of the solvent fraction during drying, and the difficulty of identifying the amorphous background, it is hard to precisely determine the crystallinity of the drying beads from the present measurements. The diffraction patterns of the cellulose-II structure on the 2D WAXS images for 1.5 wt %-H_2_O beads (shown in Figure S5c) suggest that cellulose-II structure was formed and distributed homogeneously inside the gel beads in the later drying phase.

### Nonsolvent Effect

**Figure 8:**
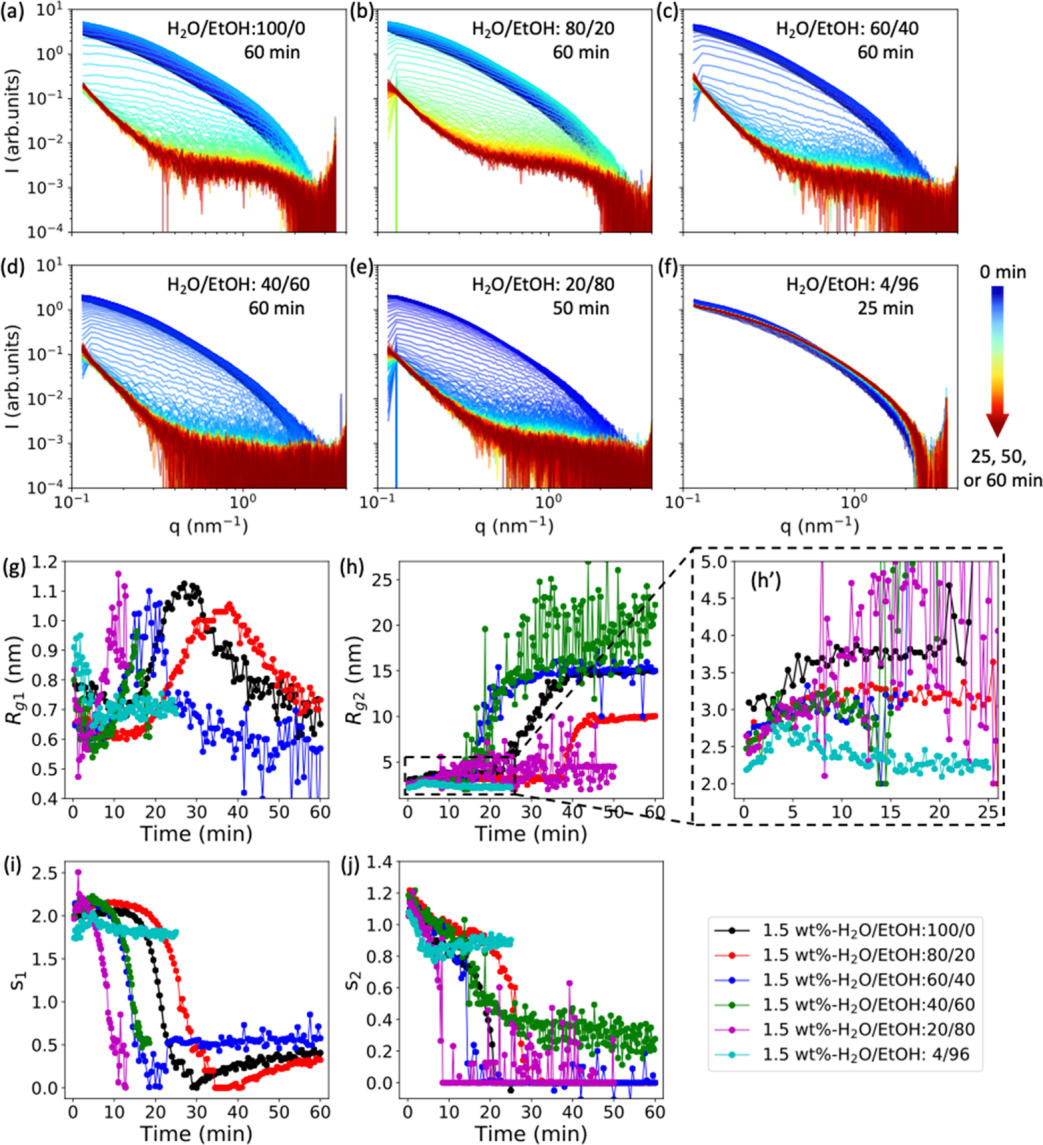
SAXS curves measured for 1.5 wt % cellulose gel beads swollen by (a) water, (b–e) different water/ethanol mixtures, and (f) ethanol, during drying process over a time of 60, 50, or 25 min, with a time step between each curve of 30 s for (a–c) and 15 s for (d–f). The color arrow bar indicates the drying time from 0 min (blue) to 25, 50, or 60 min (red). (g–j) Change in the fitted length scale (*R*_*g*1_ and *R*_*g*2_) and “dimensionality” parameters (*s*_1_ and *s*_2_) as a function of the drying time.

To investigate the effect of the nonsolvent on the drying behavior, additional SAXS measurements were performed for the 1.5 wt % cellulose gel beads swollen with different water/ethanol mixtures. The extracted 1D SAXS curves for all 1.5 wt % gel beads swollen by water, ethanol, and water/ethanol mixtures (volume ratio: 80/20, 60/40, 40/60, and 20/80) during the drying process are shown in Figure 8a–f. Before drying, the SAXS curves for all the beads are very similar, except for the 1.5 wt %-EtOH bead (Figure 8f). The gel beads in Figure 8b–e show three drying regimes, which are the same as observed in the 1.5 wt %-H_2_O bead (Figure 8a). However, in the very late drying phase, the shape of the SAXS curves for these beads changes as the ethanol fraction is increased, especially in the high *q* region: *q* > 0.5 nm^–1^ (from Figure 8b–e). In the SAXS results for water swollen beads (Figure 4), it can be seen that there is a sharp structural change for all the 1.5 wt % gel beads swollen with water/ethanol mixtures. This is verified by the fitting results plotted in Figure 8g–j and Figure S9, where a sharp increase in *R*_*g*2_ is observed for all the 1.5 wt % gel beads swollen in water/ethanol mixtures. However, for the 1.5 wt % gel bead swollen with water/ethanol (20/80), *R*_*g*2_ only slightly increases from 2.5 to 4.5 nm meaning that the aggregation size gets roughly two-times larger. It is significantly lower than for the other water/ethanol mixtures (Figure 8h), most probably due to its low water content. In the case of the 1.5 wt %-EtOH bead, which contains only 4 vol % water, *R*_*g*2_ does not increase during drying. On the basis of these results, it is suggested that, from a structural point of view, water affects the drying behavior more than ethanol. This is to be expected given the large interaction between water and cellulose.

**Figure 9:**
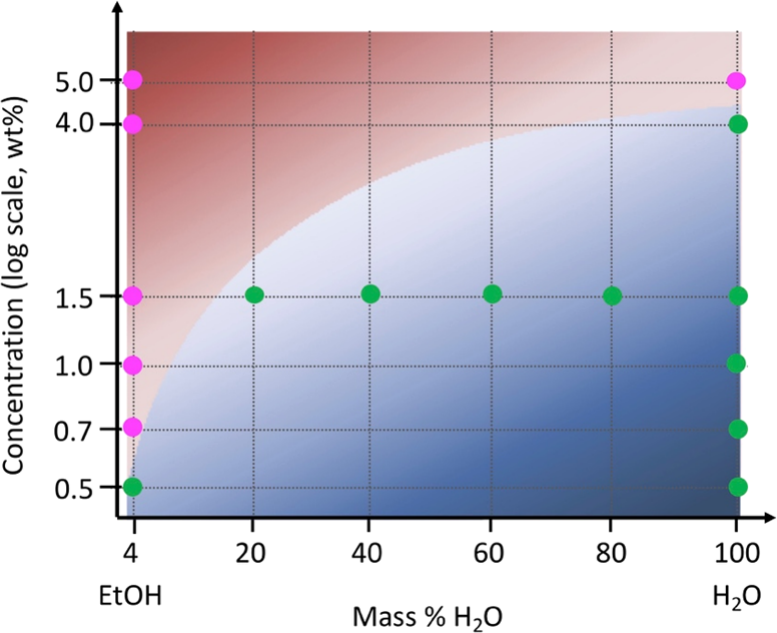
Simplified schematic illustration summarizing the structural evolution during drying of the cellulose beads based on SAXS and WAXS results. Green points represent a three-step drying process in which there is a sharp structural change, and magenta points show a two-step drying process where there is no sharp change in the cellulose structure.

On the basis of the results obtained from SAXS and WAXS measurements, a simplified schematic illustration summarizing the structural evolution of cellulose gel beads drying from water, ethanol, and water/ethanol mixtures is presented in Figure 9. Note, the applicability of this phase diagram for other nonsolvents needs to be further investigated. This phase diagram enables the quick and easy prediction of the drying behavior of regenerated cellulose gel materials swollen by water/ethanol mixtures on the macro- and micro- scale. For example, when the cellulose concentration is lower than 0.5 wt %, there will always be a structural change regardless of the ratio of the water/ethanol mixture used. When the cellulose concentration is higher than 5.0 wt %, there will never be any structural change in any of the samples, regardless of the water/ethanol ratio. However, when the cellulose concentration is between 0.5 and 5.0 wt %, the occurrence of the structural change depends on the nonsolvent composition. For example, the structure change can be observed for 1.5 wt % beads when the water fraction in the nonsolvent is greater than 20 vol %. However, more precise phase diagram with accurate phase boundary needs to be updated in feature work.

## Conclusion

The drying behaviors of regenerated cellulose gel beads with different cellulose concentrations and swollen by water, ethanol, or water/ethanol mixtures were investigated. The drying kinetics were traced by *in situ* optical microscopy, SAXS, and WAXS. From the macro scale changes in bead diameter and the micro or nano scale structural evolution during drying, a two-step or a three-step drying process is observed, depending on the cellulose concentration and the nonsolvent used. A simplified phase diagram is proposed to describe the cellulose gel beads’ drying behavior. When the cellulose concentration is lower than 0.5 wt %, a three-step drying process is observed for all the gel beads studied, regardless the ratio of water to ethanol in the nonsolvent used. A sharp structural transition corresponding to the formation of cellulose II crystalline structures occurs during the three-step drying. When the cellulose concentration is higher than 5.0 wt %, a two-step drying behavior is observed, independent of the nonsolvent composition, no structural transition occurs in these beads. The drying behavior is more complicated when the cellulose concentration is between 0.5 and 5.0 wt %. Both two-step and three-step drying behaviors can be observed depending on the water content of the nonsolvent. For example, for 1.5 wt % gel beads, a three-step drying behavior occurs when the water content is higher than 20% in the water/ethanol mixture, and a two-step drying behavior is observed when the water content is lower than 20%. The results presented herein further our understanding of the drying behavior of cellulose on a molecular level. Such advances are invaluable for the preparation of cellulose-based materials such as fibers, membranes, and adhesives. It is also of interest that crystalline cellulose II structures can be developed during drying when the regenerated aggregate structures are able to reorganize at lower concentrations of the cellulose.

## Methods and Experimental Section

### Materials

Domsjö dissolving pulp fibers (Domsjö Fabriker AB, Sweden) are the raw material used to prepare the cellulose/DMAc/LiCl solutions and gel beads. The fibers from this dissolving pulp contain 96% glucose.^21,44^ Lithium chloride (LiCl, puriss p.a., anhydrous ≥99%), *N*,*N*-dimethylacetamide (DMAc, puriss p.a., ≥ 99.5%), and ethanol (EtOH, 96 vol %) were purchased from Sigma-Aldrich. All chemicals were used without further purification.

### Preparation of Cellulose/LiCl/DMAc Solution

To make the cellulose gel beads, cellulose/LiCl/DMAc solutions with different cellulose concentrations were first prepared according to a previously established protocol.^20,21,24,33,45,46^ The dissolving grade fibers were prewashed with deionized water to remove metal ions and dissolved colloidal substances (carbohydrates, lignin, and extractives). Water saturated dissolving fibers containing 5.0 g of dry mass were solvent exchanged with ethanol and then DMAc through multiple washing/filtration steps. The solvent-exchange was performed over 2 days for each solvent, the solvent being changed at least twice a day, using 150 mL each time. After the solvent exchange, 100 mL of DMAc was heated to 105 °C for 20 min in an oil bath, and 7 g of LiCl was heated in an oven at 105 °C for 30 min to remove entrapped water. The dehydrated LiCl was added to the heated DMAc and then allowed to cool to 65 °C at which point the DMAc saturated pulp was added. After stirring overnight, the 5.0 wt % cellulose/LiCl/DMAc solution was obtained. The same procedure was performed to prepare 1.5 wt % cellulose/LiCl/DMAc solution. Part of 5.0 wt % solution was diluted to 4.0 wt % and part of 1.5 wt % solution was diluted to 1.0, 0.7, and 0.5 wt % with DMAc.

### Preparation of Cellulose Swollen Beads

The 6 different concentrated cellulose/DMAc/LiCl solutions were precipitated dropwise into nonsolvent baths (ethanol, 96 vol %), where the cellulose solution drop solidified into the initial cellulose gel beads, as depicted in Figure S1. The precipitation was performed using an infusion pump (Harvard Apparatus, Holliston, MA, model PHD 2000). The prepared beads were left to equilibrate for 24 h in the ethanol baths. Then the beads prepared from 5.0, 4.0, 1.0, 0.7, and 0.5 wt % solutions were divided into two fractions: (i) beads washed with Milli-Q water and (ii) beads washed with ethanol. Both fractions were washed with their respective solvents for at least 7 days to ensure a proper removal of the DMAc/LiCl. Beads obtained by washing with water or ethanol (96 vol %) are labeled water swollen beads (5.0 wt %-H_2_O, 4.0 wt %-H_2_O, 1.0 wt %-H_2_O, 0.7 wt %-H_2_O, and 0.5 wt %-H_2_O) and ethanol swollen beads (5.0 wt %-EtOH, 4.0 wt %-EtOH, 1.0 wt %-EtOH, 0.7 wt %-EtOH, and 0.5 wt %-EtOH), respectively.

For the beads prepared from the 1.5 wt % solution, they were divided into six fractions and washed with Milli-Q water, Milli-Q water/ethanol mixtures (volume ratio: 80/20, 60/40, 40/60, 20/80) or ethanol for at least 7 days. These were labeled as 1.5 wt %-H_2_O, 1.5 wt %-H_2_O/EtOH(80/20), 1.5 wt %-H_2_O/EtOH(60/40), 1.5 wt %-H_2_O/EtOH(40/60), 1.5 wt %-H_2_O/EtOH(20/80), and 1.5 wt %-EtOH.

### Preparation of Cellulose Dry Beads

To observe the morphology of the initial cellulose gel beads with a scanning electron microscope (SEM), ethanol swollen beads were chosen to prepare the dry samples using a critical point drying method (CPD), during which the capillary forces between the vapor, liquid, and solid cellulose is theoretically excluded.^47^ The ethanol swollen cellulose beads were solvent exchanged to pure ethanol over 2 days refreshing the solvent three times per day. The beads were then placed in the CPD chamber (Autosamdri-815, Tousimis, USA) and liquid carbon dioxide was injected into the chamber under a pressure of ∼50 bar for solvent exchange from ethanol to CO_2_. The conditions of the chamber were then brought above the CO_2_ critical point, to ∼100 bar and 36 °C, after which the chamber was depressurized and the CO_2_ evaporated.

### Field Emission Scanning Electron Microscope (FE-SEM)

The interior morphologies of the CPD dried cellulose beads were characterized using a S-4800 field emission scanning electron microscope (FE-SEM) (Hitachi, Tokyo, Japan) operating at high vacuum. CPD dried beads were cut and glued onto a conductive carbon tape on the sample holder and then coated with Pt/Pd in a Cressington 208 HR sputter coater (Cressington Scientific Instruments, Watford, UK) for 20 s to limit sample charging during imaging.

### Optical Microscope

An optical camera (AM7013MZT, Dino-Lite Premier Digital Microscope) was used to monitor the diameter of the cellulose gel beads throughout the drying process at 26 °C and 33% RH (the same conditions used in the SAXS/WAXS measurements). To be consistent with SAXS/WAXS measurements, Kapton tape (part number: 42–020–0016) was used as the substrate for this characterization as well.

### Small-Angle/Wide-Angle X-ray Scattering (SAXS/WAXS)

*In situ* SAXS/WAXS characterization for drying cellulose gel beads was performed at Forschungszentrum Jülich, Germany. The X-ray source is a D2-MetalJet (Excillum) with a liquid metal anode operating at 70 kV and 3.57 mA with Ga–Kα radiation (wavelength λ = 0.1314 nm), providing a brilliant and narrow beam (<100 μm). The X-ray beam was further focused with a focal length of 55 cm, using a specially made X-ray optic (Xenocs) to provide a very narrow (0.15 × 0.15 mm^2^) and intense beam at the sample position. The scattering data were acquired with a position-sensitive detector (PILATUS 300 K, Dectris) with a pixel size of 172 μm. After calibration with silver behenate, the sample-to-detector distances were set to 1107 mm and 152 mm for SAXS and WAXS measurements, respectively. The cellulose beads were adhered to the Kapton tape surface, which prevents the beads from sliding when the sample holder was placed in the vertical position. Individual 2D scattering patterns were recorded for water and ethanol swollen beads. The SAXS scans took 30 s each and the WAXS took 15 s to scan. After radial integration, the background scattering of the Kapton tape was scaled and subtracted for each curve to obtain more accurate data. Scaling was completed to account for the change in X-ray transmission that occurs from bead shrinking during drying.

## Supporting Information Available

SEM images of different CPD dried cellulose gel beads, microscopic images of cellulose gel beads before and after drying, *in situ* SAXS/WAXS experiments and 2D SAXS/WAXS patterns, SAXS profiles and the corresponding fitting curves, parameters extracted from the SAXS fitting curves (PDF)
