# Supplementary material for: Understanding the Drying Behavior of Regenerated Cellulose Gel Beads: The Effects of Concentration and Nonsolvents
Source: ACS Nano. 2022 Feb 1;16(2):2608–20. doi: 10.1021/acsnano.1c09338 (PMC8867908; doi:10.1021/acsnano.1c09338)
Supplement: Supplementary file 1 — nn1c09338_si_001.pdf [file nn1c09338_si_001.pdf]

Supporting Information

Understanding the Drying Behavior of Regenerated Cellulose Gel Beads: The Effects of Concentration and Nonsolvents

Hailong Li[1,2], Margarita Kruteva[3], Martin Dulle[3], Zhen Wang[1], Katarzyna Mystek[1], Wenhai Ji[4], Torbjörn Pettersson[1,5]*, and Lars Wågberg[1,5]**

[1]Department of Fibre and Polymer Technology, KTH Royal Institute of Technology, Teknikringen 58, SE-100 44 Stockholm, Sweden

[2]Department of Physics, AlbaNova University Center, Stockholm University, 10691 Stockholm, Sweden

[3]Jülich Centre for Neutron Scattering and Biological Matter (JCNS-1/IBI-8), Forschungszentrum Jülich GmbH,Wilhelm-Johnen-Straße, D-52425 Jülich, Germany

[4]Deutsches Elektronen-Synchrotron (DESY), Notkestr. 85, 22607 Hamburg, Germany

[5]Wallenberg Wood Science Centre, Department of Fibre and Polymer Technology, KTH Royal Institute of Technology, Teknikringen 56, 10044 Stockholm, Sweden

*Email: haili@kth.se, torbj@kth.se, wagberg@kth.se

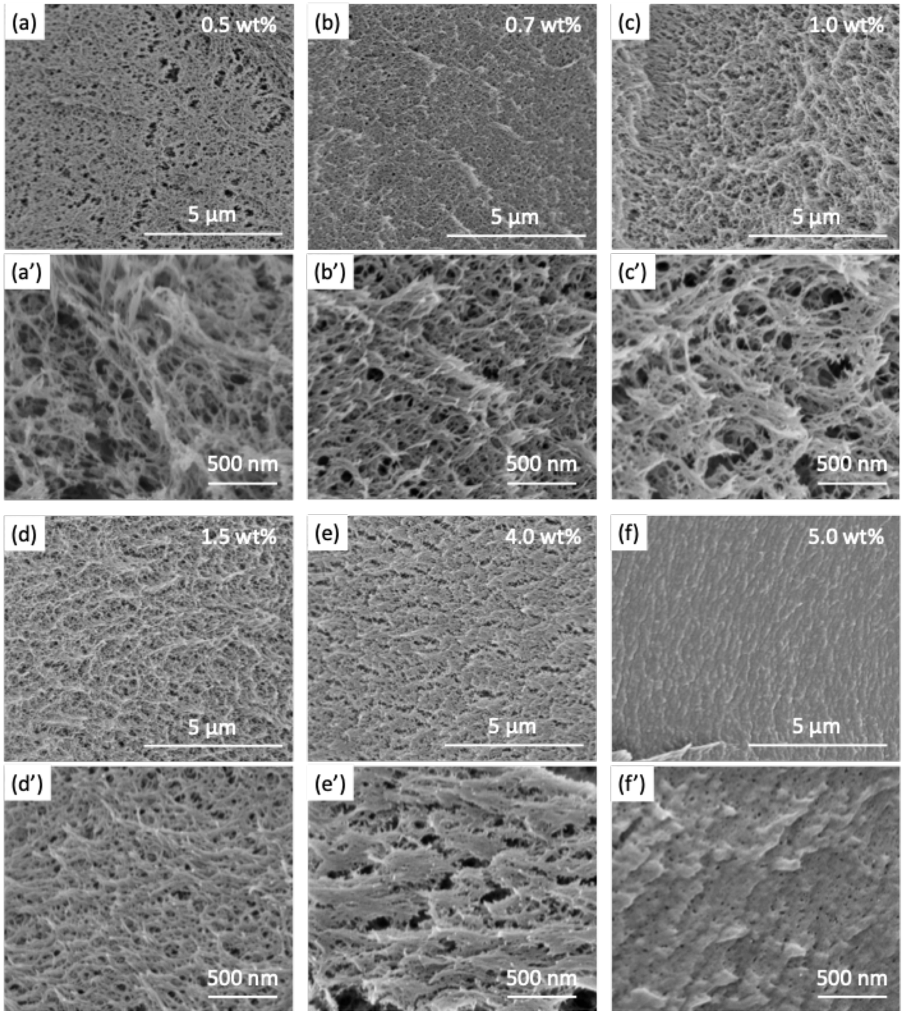

Figure S1. SEM images of the interior of CPD dried water swollen cellulose beads prepared using different cellulose concentrations: (a, a') 0.5 wt%-H_20, (b, b') 0.7 wt%-H_20, (c, c') 1.0 wt%-H_20, (d, d') 1.5 wt%-H_20, (e, e') 4.0 wt%-H_20, and (f, f') 5.0 wt%-H_20. (a-g) are lower magnification SEM images and (a'-g') are higher magnification SEM images.

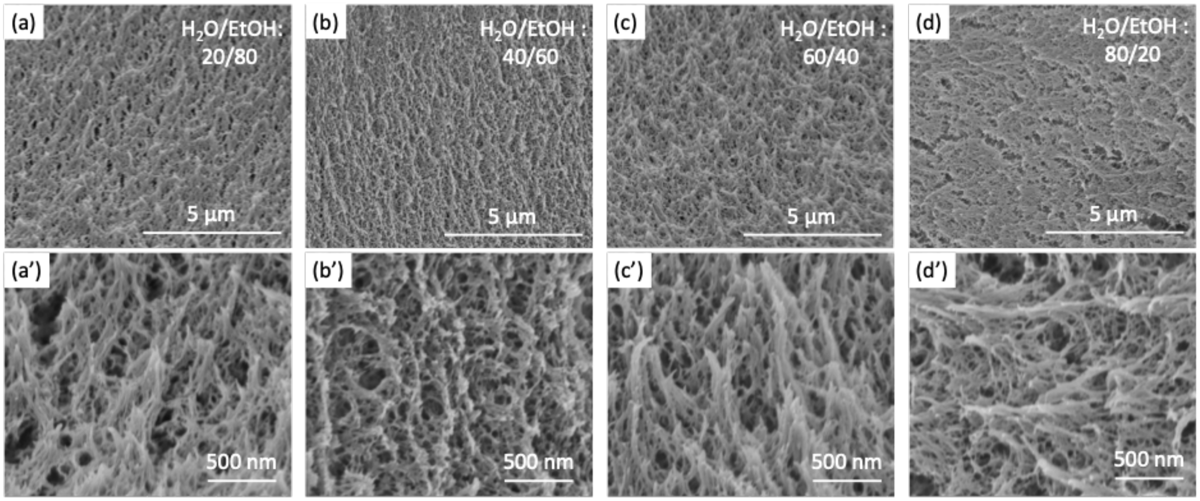

Figure S2. SEM images of the interior of CPD dried 1.5 wt% cellulose beads swollen by water/ethanol mixtures: (a, a') H_20/EtOH: 20/80, (b, b') H_20/EtOH: 40/60, (c, c') H_20/EtOH: 60/40, and (d, d') H_20/EtOH: 80/20. (a-d) are lower magnification SEM images and (a'-d') are higher magnification SEM images.

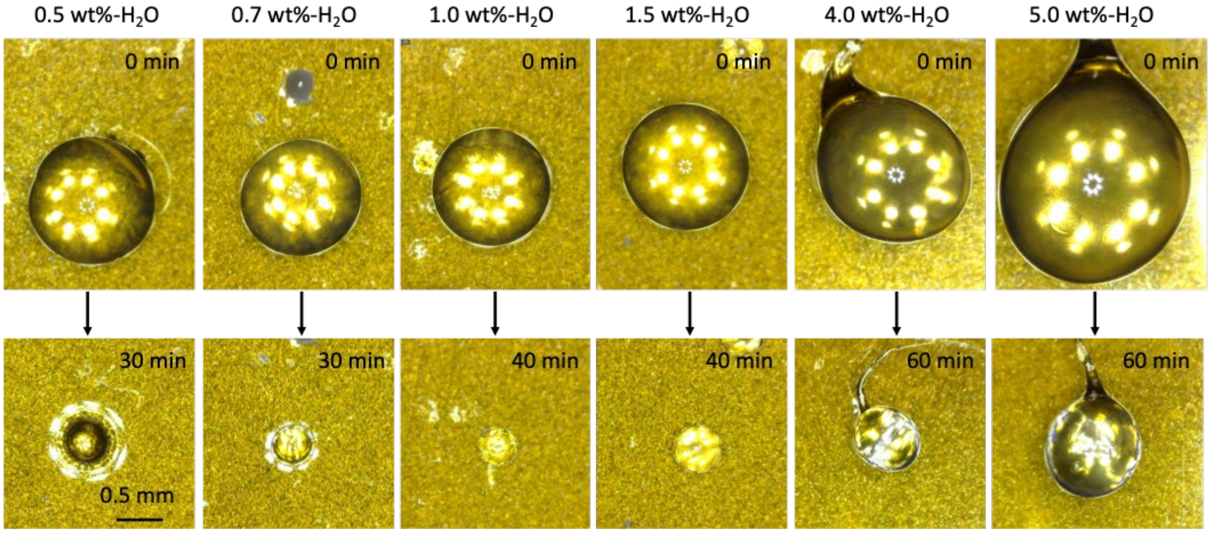

Figure S3. Top-view optical microscope images of 0.5, 0.7, 1.0, 1.5, 4.0, and 5.0 wt%-H_2O beads before drying (0 min) and after drying for a defined period of time. The scale bar corresponds to a length of 0.5 mm for all the images.

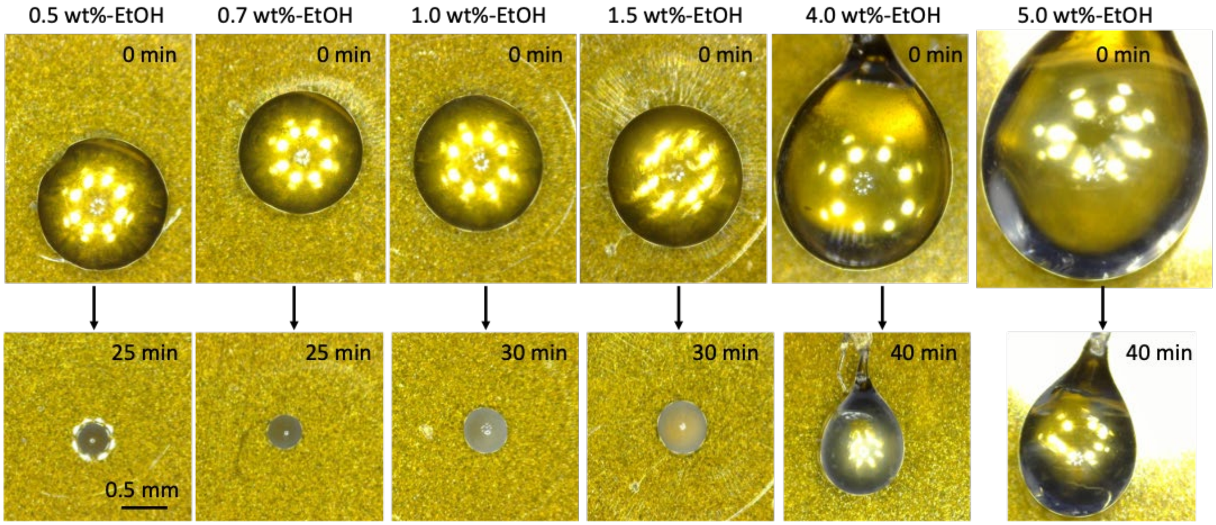

Figure S4. Top-view optical microscope images of 0.5, 0.7, 1.0, 1.5, 4.0, and 5.0 wt%-EtOH beads before drying (0 min) and after drying for a defined period of time. The scale bar corresponds to a length of 0.5 mm for all the images.

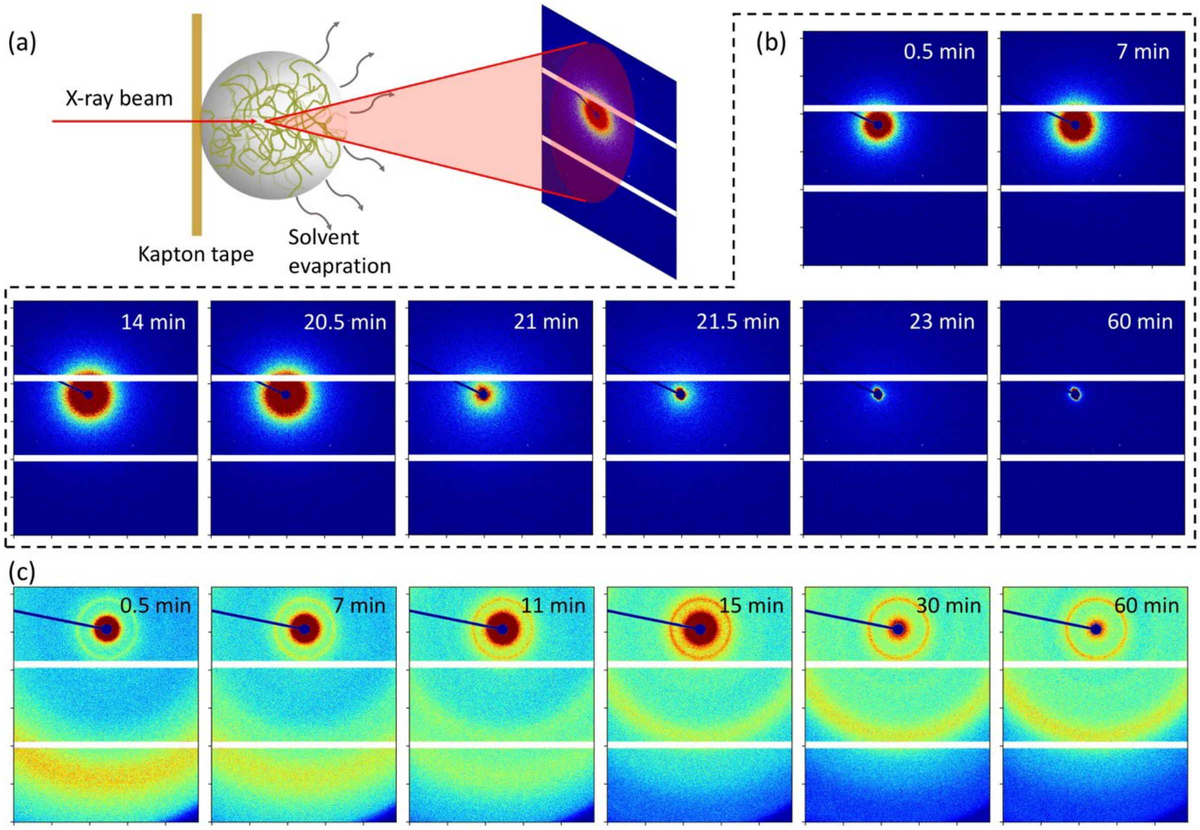

Figure S5. (a) Schematic illustration of the in situ SAXS/WAXS measurements during drying of the wet beads on Kapton tape at 26 ºC and 33% RH. Representative 2D SAXS (b) and 2D WAXS (c) patterns for a 1.5 wt%-H_2O bead. Each pattern was measured for 30 s.

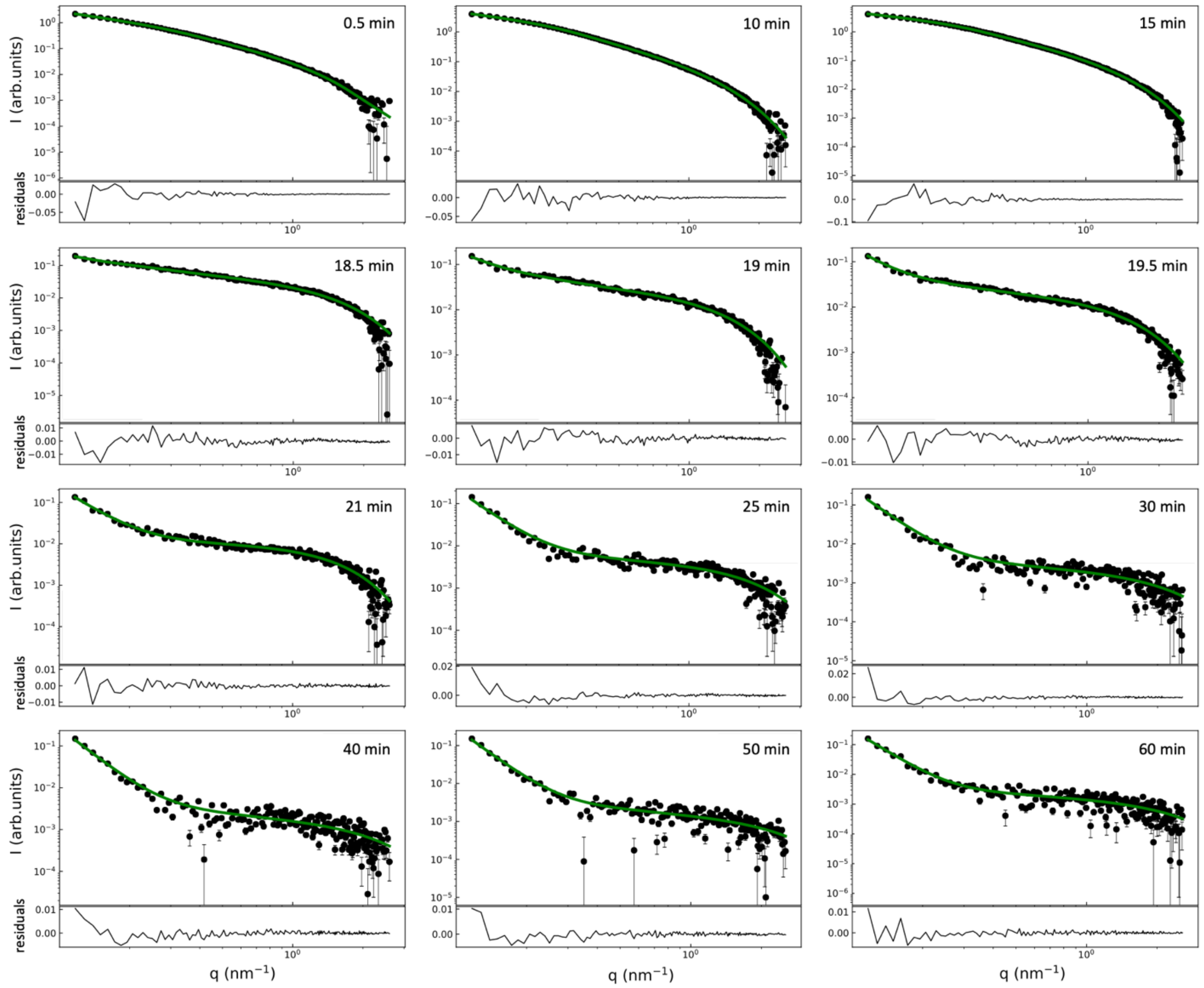

Figure S6. SAXS data for a 1.0 wt%-H_2O bead after drying for 0.5, 5, 10, 18.5, 19, 19.5, 21, 25, 30, 40, 50 and 60 minutes (black points) and the corresponding Guinier-Porod model fitting curves in green, the residuals from the fitting are presented as a black line below each data point.

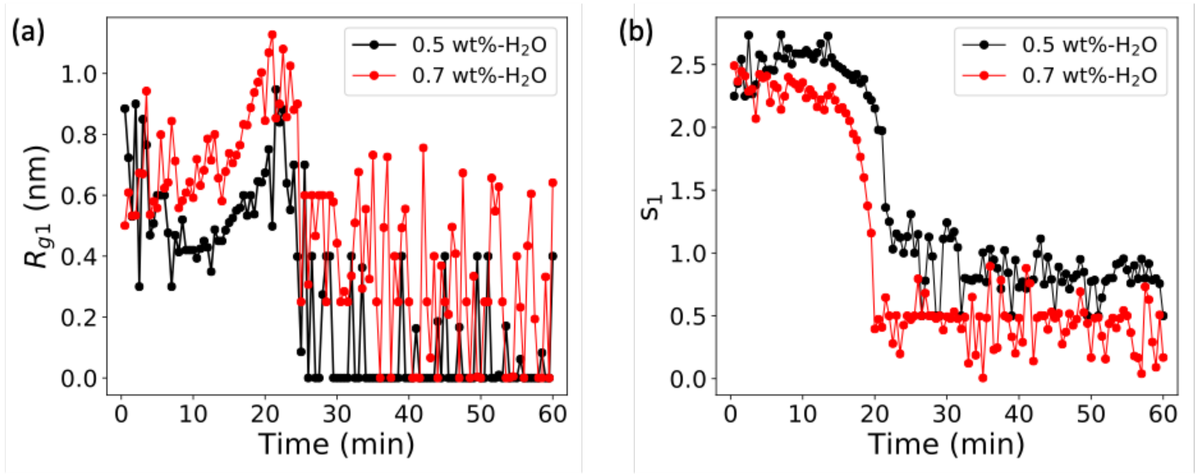

Figure S7. The change in the fitted length scale R_{g1} (a) and 'dimensionality' parameter s_1 (b) as a function of the drying time for 0.5 wt% and 0.7 wt%-H_2O beads.

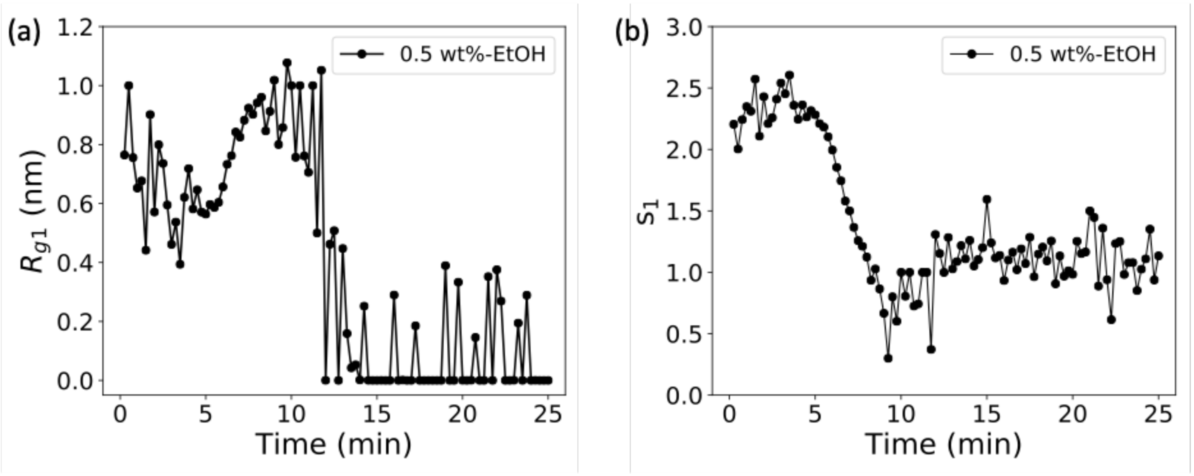

Figure S8. The change in the fitted length scale R_{g1} (a) and ‘dimensionality’ parameter s_1 (b) as a function of the drying time for a 0.5 wt%-EtOH bead.

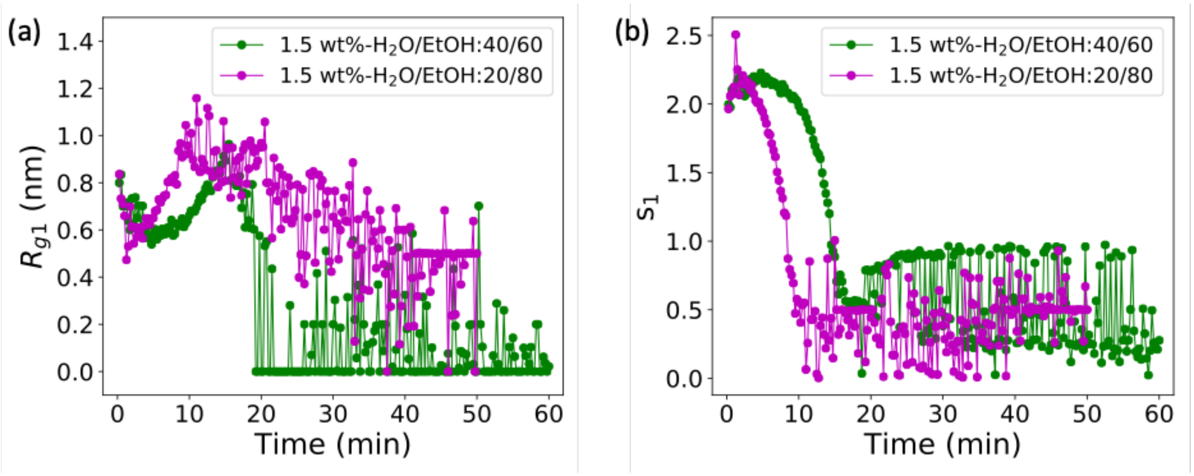

Figure S9. The change in the fitted length scale R_{g1} (a) and ‘dimensionality’ parameter s_1 (b) as a function of the drying time for 1.5 wt%-H_2O/EtOH:40/60 and 1.5 wt%-H_2O/EtOH:20/80 beads.
